# PCR cloning of a histone H1 gene from *Anopheles stephensi *mosquito cells: comparison of the protein sequence with histone H1-like, C-terminal extensions on mosquito ribosomal protein S6

**DOI:** 10.1186/1471-2164-6-8

**Published:** 2005-01-24

**Authors:** Yongjiao Zhai, Ann M Fallon

**Affiliations:** 1 Department of Entomology, University of Minnesota, 1980 Folwell Ave., St. Paul, MN, 55108 USA

## Abstract

**Background:**

In *Aedes *and *Anopheles *mosquitoes, ribosomal protein RPS6 has an unusual C-terminal extension that resembles histone H1 proteins. To explore homology between a mosquito H1 histone and the RPS6 tail, we took advantage of the *Anopheles gambiae *genome database to clone a histone H1 gene from an *Anopheles stephensi *mosquito cell line.

**Results:**

We designed specific primers based on RPS6 and histone H1 alignments to recover an *Anopheles stephensi *histone H1 corresponding to a conceptual *An. gambiae *protein, with 92% identity. Southern blots suggested that *Anopheles stephensi *histone H1 gene has multiple variants, as is also the case for histone H1 proteins in Chironomid flies.

**Conclusions:**

Histone H1 proteins from *Anopheles stephensi *and *Anopheles gambiae *mosquitoes share 92% identity to each other, but only 50% identity to a *Drosophila *homolog. In a phylogenetic analysis, *Anopheles*, *Chironomus *and *Drosophila *histone H1 proteins cluster separately from the histone H1-like, C-terminal tails on RPS6 in *Aedes *and *Anopheles *mosquitoes. These observations suggest that the resemblance between histone H1 and the C-terminal extensions on mosquito RPS6 has been maintained by convergent evolution.

## Background

Ribosomal protein (RP) S6 is a phosphorylated protein that resides on the small subunit of eukaryotic ribosomes. Phosphorylation occurs on a cluster of five serine residues near the C-terminal end of the protein. Although details remain unclear, the phosphorylation state of RPS6 is believed to influence translational efficiency of some mRNAs [1], possibly mediated by direct contact between RPS6 and the 28S rRNA in the large subunit. RPS6 has also been implicated in ribosome biogenesis, and is thought to play a conserved role in the initiation of protein synthesis [2].

In *Aedes aegypti *and *Aedes albopictus *mosquitoes, the RPS6 protein is ~17 kDa larger than its *Drosophila *homolog, and on polyacrylamide gels, it migrates as the largest protein from the small ribosomal subunit. *Ae. aegypti *and *Ae. albopictus *RPS6 cDNAs encode an approximately 100 amino acid extension at the C-terminal end of the protein. The extension is particularly rich in lysine, alanine and glutamic acid, and most closely resembles the sequence of histone H1 proteins from diverse sources [3].

Because RPS6 is thought to have regulatory function(s) in a variety of cell signaling pathways [2], we were surprised to uncover this difference between mosquito and *Drosophila *RPS6 proteins. We have recently shown that RPS6 protein isolated from ribosomal subunits retains its histone H1-like tail [4]. Thus, unlike the case with the ubiquitinated ribosomal protein S27a in the rat [5], the histone tail is not removed from the mosquito ribosomal protein prior to ribosome assembly.

*RpS6 *cDNA from an *Anopheles stephensi *cell line encodes an approximately 170 amino acid histone H1-like C-terminal extension, and in silico analysis reveals a similar modification encoded by the *rpS6 *gene in *Anopheles gambiae*. In both *Aedes *and *Anopheles *mosquitoes, the C-terminal extension was completely encoded in Exon 3, directly contiguous with upstream open reading frame encoding the series of serines that may be phosphorylated [4]. Anopheline mosquitoes are believed to be ancestral to the Culicidae, which includes the genera *Aedes *and *Culex *[6]. Thus, to a first approximation, we infer that the longer tail in *Anopheles *mosquitoes represents the ancestral state, and that the RPS6 tail has been lost in the higher Diptera, which include *D. melanogaster*.

Although mosquito RPS6 tails in general resemble histone H1 proteins, their divergence between *Aedes *and *Anopheles *mosquitoes was high, relative to the conventional portion of the RPS6 coding sequence. Because histone H1 is the most variable of the histone proteins, and functions as a linker, rather than as a component of the histone octamer, we set out to clone a cDNA encoding a bona fide histone H1 protein from an *An. stephensi *cell line. In a phylogenetic comparison, the *An. stephensi *histone H1 protein clusters with homologs from *Drosophila *and *Chironomus*, rather than with RPS6 histone H1-like tails from mosquitoes. These results indicate that the histone H1-like tails on mosquito RPS6 proteins are evolving independently of conspecific histone H1 proteins.

## Results

### Design of PCR primers

The gene encoding *Drosophila melanogaster *histone H1 spans 1204 nucleotides, and encodes a 256 amino acid protein in a single exon [7]. There is a single recorded *His1 *allele in *Drosophila *[8], while multiple histone H1 variants have been described in Chironomid flies [9-11]. When the deduced sequence of the *Drosophila *histone H1 protein (Accession NM_058232) was compared to the *Anopheles gambiae *genome using the program BLAST [12] on the NCBI website (National Center for Biotechnology Information; ), we obtained 5 accessions with E values ranging from 3e-35 to 8e-43, distributed on mosquito chromosomes 2 and 3. Upon further examination, we noted that XP_314184 and XP_314186 (chromosome 2) corresponded to the same protein. Two additional histone H1 candidates (XP_311486 and XP_309451) were encoded on chromosome 3. These three conceptual *Anopheles *proteins shared 70–80% identity to one another, and about 50% identity to the *Drosophila *H1 protein sequence. In the EST-other database, we found a single uninformative match to an unidentified *An. gambiae *entry (dbEST id = 11236311), with the relatively modest E value of 0.055. Histone H1 sequences from *Aedes *mosquitoes are not yet in existing databases.

The 50% identity between *Drosophila *and *Anopheles *histone H1 proteins was relatively low, compared to approximately 80% amino acid identity between *Drosophila *and *Anopheles *RPS6, exclusive of the histone-H1-like tail in the mosquito protein. The *Drosophila *H1 histone was also ~50% identical to that from *Chironomus thummi*, a fly closely related to mosquitoes in the infraorder/superfamily Culicomorpha [13].

**Figure 1:**
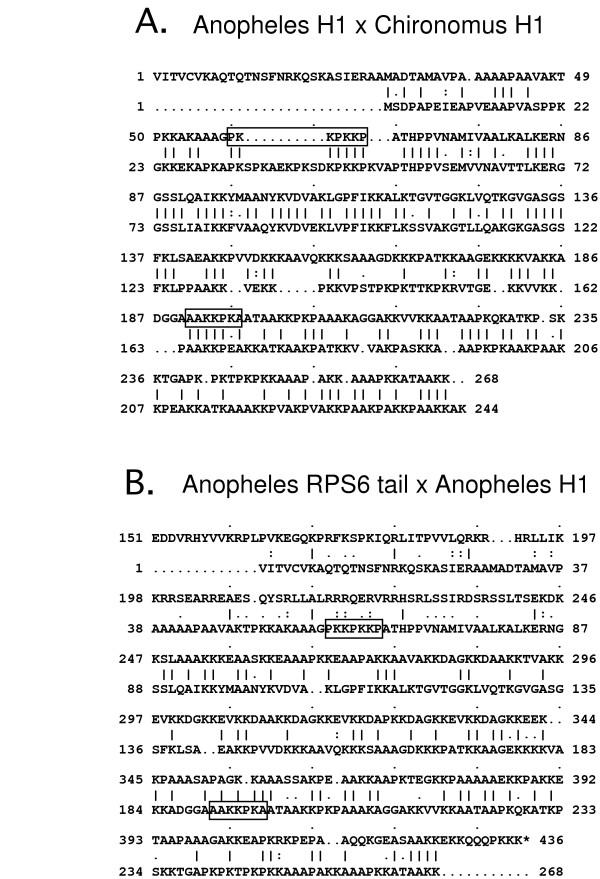
Primer design. To design primers, we aligned an *An. gambiae *putative histone H1 candidate XP_311486 (Panel A, top) with a histone H1 protein (Q07134; Panel A, bottom) from *C. thummi*. Boxed residues were chosen for design of primers, according to the *An. gambiae *nucleotide sequence. Panel B shows these primer residues aligned between the *An. stephensi *RPS6 tail (top), and the putative *Anopheles gambiae *histone H1 (bottom). Vertical bars designate identities.

**Figure 2:**
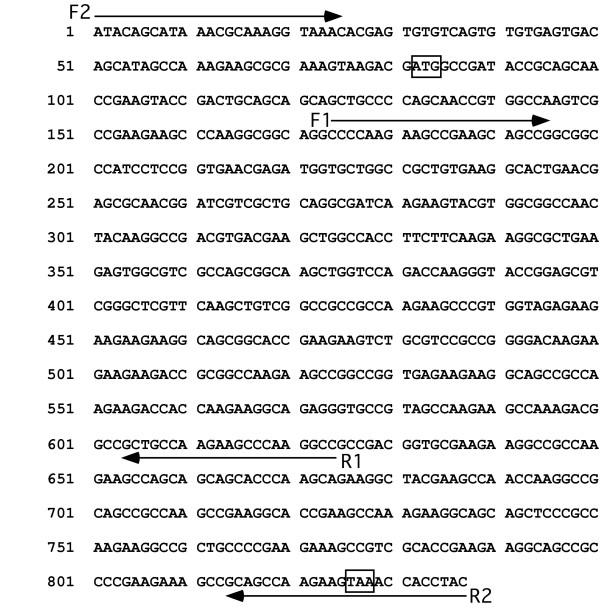
Sequence of *An. stephensi *histone H1 gene. The positions of internal primers F1 and R1, and primers F2 and R2 are designated by arrows. The ATG start codon and TAA stop codon are boxed.

To design primers that would amplify a histone H1 gene, and not the histone H1-like tail in mosquito *rpS6*, we aligned one of the *An. gambiae *H1 candidate proteins (XP_311486) to a histone H1 protein from *C. thummi*, and examined the alignment for precise matches (Fig. 1A) that did not match well in a separate alignment of the *An. gambiae *histone H1 protein with the *An. gambiae *RPS6 tail (Fig. 1B). The forward primer (F1) corresponded to amino acids PKKPKKP in *An. gambiae*, and a reverse primer (R1) corresponded to residues AAKKPKA (Fig. 2).

### Recovery of *An. stephensi *histone H1 gene

We used F1 and R1 primers with *Hin*dIII-digested genomic DNA from *An. stephensi *cells to obtain an approximately 450 bp PCR product, which was sequenced and verified to encode a histone H1 protein. The 5-end of the gene, which extended 81 nucleotides upstream of the ATG start codon, was obtained using primer R1 with the GeneRacer kit (Invitrogen, Carlsbad, CA), with total RNA as the template. The absence of a poly(A) tail on histone mRNAs required an unconventional strategy to obtain the 3'-end of the coding sequence. First, we used *Hin*dIII-digested genomic DNA template, with a primer based entirely on the 3'-UTR of *An. gambiae *XP_314184, without success. When we designed a second primer (R2, in Fig. 2) extending from the 3'-UTR through the TAA stop codon and into the coding region, we obtained the 3'-end of the coding sequence. Finally, primers F2 and R2 (Fig. 2) were used to verify the complete nucleotide sequence.

### Southern blots with *An. stephensi *genomic DNA

**Figure 3:**
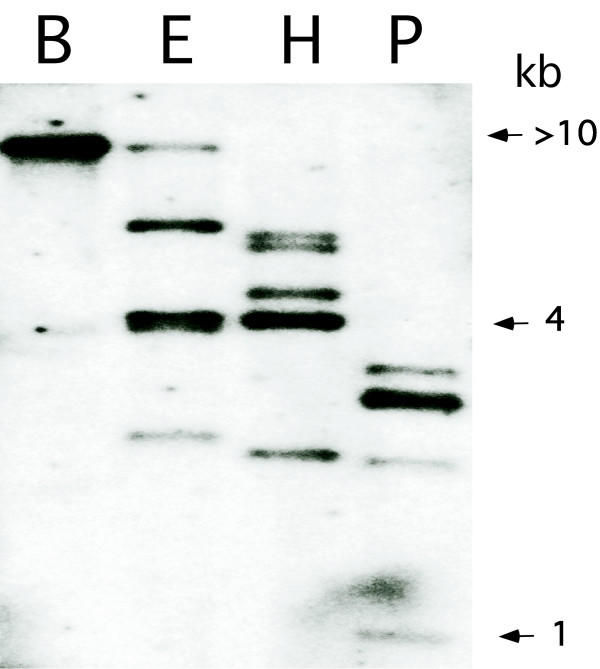
Southern blot of *An. stephensi *genomic DNA hybridized to the *An. stephensi *histone H1 probe. DNA was digested with *Bam*HI (B), *Eco*RI (E), *Hin*dIII (H) and *Pvu*I (P). Positions of size markers are shown at right.

The likelihood that the mosquito genome contains multiple histone H1 gene variants is consistent with the multiple H1 variants that have been described in *Chironomus *[9-11] and eight histone H1 subtypes that have been described in mammals [14,15]. When we used the *An. stephensi *cDNA to probe Southern blots of genomic DNA digested with various restriction enzymes with 6 bp recognition sites, most enzymes gave multiple bands, with the notable exception of *Bam*HI, which hybridized to a single band longer than 10 kb (Fig. 3). Based on the observation that *D. melanogaster *H1, H2A, H2B, H3 and H4 histone genes are organized in approximately one hundred 5 kb repeats per haploid genome [16], the large *Bam*HI fragment from *An. stephensi *may be a starting point for recovery of a complete cluster of the *An. stephensi *histone gene family.

**Figure 4:**
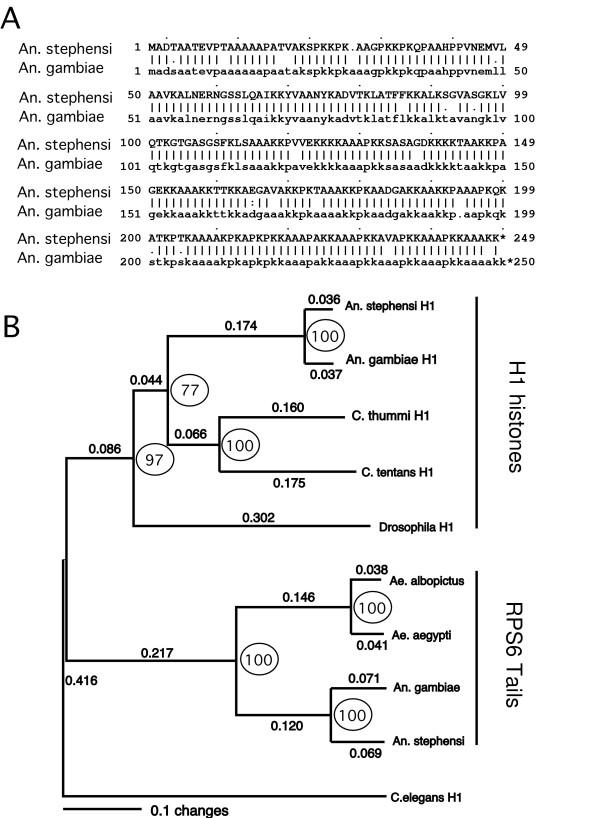
Comparison of mosquito histone H1 proteins and RPS6 histone H1-like tails. Panel A shows the alignment of the experimentally-determined *An. stephensi *histone H1 amino acid sequence, compared to *An. gambiae *conceptual protein XP_314184. Panel B shows a phylogram produced in PAUP* by neighbor joining, with the nematode *C. elegans *histone H1-like protein 2 (AAM44399) designated as the outgroup. The alignment includes histone H1 proteins from various Diptera, and the known histone H1-like tails on mosquito RPS6. Values on the horizontal lines indicate branch lengths, defined as the fraction of substitutions between the nodes that define the branch. Bootstrap values based on 1000 replicates are shown within circles. A single tree with identical topology was obtained with the optimality criterion set to parsimony.

The *An. stephensi *nucleotide sequence (GenBank accession # AY672907) matched *An. gambiae *histone H1 candidates on chromosomes 2 and 3 with an E value of 0.0. In addition, 6 unmapped sites also had E values of 0.0. A final two sites had E values of 4e-170 and 3e-127. The deduced *An. stephensi *protein sequence was 92% identical to *An. gambiae *protein XP_314184 on chromosome 2 (Fig. 4A). A similar level of identity was obtained with *An. gambiae *XP_309451 on chromosome 3, but the alignment required introduction of a 58 amino acid gap in the shorter (190 residue) deduced *Anopheles gambiae *protein (not shown). Identity with *An. gambiae *XP-311486 was 79%. Based on these criteria, we have cloned the *An. stephensi *homolog of *An. gambiae *XP_314184.

### Comparisons of histone H1 proteins with mosquito RPS6 C-terminal extensions

The identity between *Drosophila *and *Anopheles *(or *Drosophila *and *Chironomus*) histone H1 proteins was only 50%. This divergence undoubtedly reflects the ~250 million years [6] separating Nematoceran from Cyclorrhaphan diptera. In this study, we were interested in comparing mosquito histone H1 proteins to the histone H1-like tails of mosquito RPS6. Fig. 4B shows a neighbor-joining analysis in which we compared protein sequences from *Aedes *and *Anopheles *RPS6 histone H1-like tails, exclusive of the conventional RPS6 protein sequence, with histone H1 proteins from the nematode *Caenorhabditis elegans *(AAM44399), the closely-related flies *Chironomus thummi *(Q07134) and *Chironomus tentans *(AAB62239), *Drosophila*, and the *Anopheles gambiae *and *Anopheles stephensi *homologs (Fig. 4A). With the *C. elegans *sequence designated as the outgroup, the phylogram shows that the RPS6 tails cluster into a distinct group relative to the Dipteran histone H1 proteins. Circled values indicate bootstrap values based on 1000 replicates. When the analysis was repeated with the optimality criterion set to parsimony, we obtained a tree with the same topology, with the 77% value shown in Fig. 4B reduced to 59%, and the 97% value reduced to 94%. The 100% values remained unchanged.

**Figure 5:**
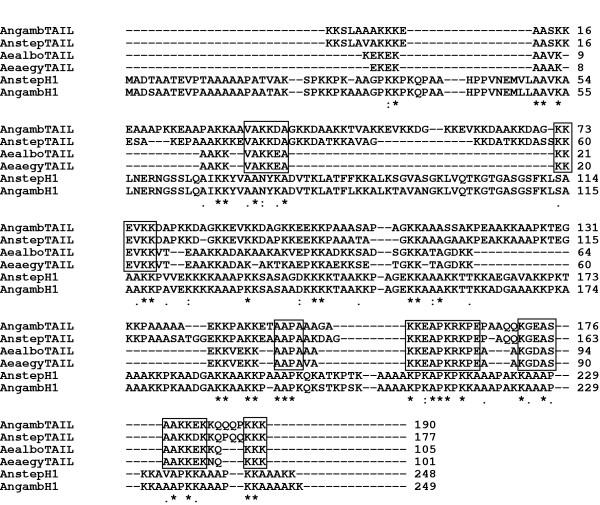
Alignment of mosquito RPS6 tails with mosquito histone H1 proteins. Angam (CAD89874), *An. gambiae*; Anstep (AY237124), *An. stephensi*; Aealbo (Q9U762), *Ae. albopictus*; Aeaegy (Q9U761), *Ae. aegypti*. The alignment was produced with ClustalX (version 1.83), using default settings. Indicators of consensus residues are shown below the alignment. Boxes in the top four entries indicate identities (aside from D, E substitutions) shared by the mosquito RPS6 tails.

In an alignment of mosquito RPS6 tails with the *Anopheles *H1 histones (Fig. 5), we note that while some degree of identity covers the entire histone H1 protein, the C-terminal half of the H1 histone has a higher proportion of identities to the RPS6 tail, as indicated by the distribution of consensus residues. Within the RPS6 tails, however, the boxed motifs:VAKK(D/E)A, KKEVKK, AAPA, KKEAPKRKPE, KG(D/E)ASAAK(E/D) are shared by all four mosquitoes. In contrast, the additional amino acids in the *Anopheles *RPS6 tails, which are represented by gaps in the *Aedes *sequences (Fig. 5), did not show regions of homology with *Anopheles *histone H1.

## Discussion

An important rationale for cloning an *An. stephensi *histone H1 was to compare its sequence to the histone H1-like tails on mosquito RPS6 ribosomal proteins. Our choice of an *Anopheles *histone H1 was based on the existing database for *An. gambiae*, the observation that the tail in *Anopheles *RPS6 is nearly twice as long as that in *Aedes *RPS6 proteins [4], and evidence that the genus *Anopheles *is ancestral to *Aedes *[6]. Because putative homologies to *Drosophila *histone H1 protein could be recovered as conceptual translation products from the *An. gambiae *database, we used these sequences to design primers that would discriminate between an *An. stephensi *histone H1 gene, and the histone H1-like extension in *An. stephensi *RPS6. Because the *Drosophila *gene was encoded in a single exon, and the histone message was unlikely to be polyadenylated [14], we used genomic DNA from *An. stephensi *as a template for our PCR reaction.

The gene we recovered had more than 90% identity to XP_314184 in *An. gambiae*. The proteins differed in length by a single amino acid residue, and showed 92 % identity. When we analyzed RPS6 tails and histone H1 genes, we found that the Dipteran histone H1 proteins and the RPS6 tails each fell into distinct groups, suggesting that in present-day mosquitoes, these proteins are evolving independently. Although these data are consistent with the possibility that present-day histone H1 proteins and the histone H1-like tails on mosquito RPS6 protein share a common ancestral gene, the histone tails seem to be evolving independently in the two mosquito genera, and have changed more rapidly than the conventional portion of mosquito RPS6 proteins.

Because RPS6 is considered an important functional component of the ribosome, it seems surprising that a histone H1-like tail occurs at the C-terminal end of this particular protein. However, histone H1-like tails have been reported at the N-terminus of *Drosophila melanogaster *ribosomal proteins L22 and L23a [17]. The *An. gambiae *homolog of *D. melanogaster *L23a also contains an N-terminal histone-like extension. The N-terminal tails of *Drosophila *L22 and L23a were found in an effort to identify proteins that interact with poly (ADP-ribose) polymerase (PARP). In future studies, we plan to explore whether the histone H1-like tail undergoes posttranslational modification, and whether it plays a functional role in ribosome biogenesis, perhaps through the activity of PARP.

### Experimental procedures

#### Mosquito cells and culture conditions

We used the ASE-IV *Anopheles stephensi *mosquito cell line [18], which was adapted to Eagle's minimal medium, supplemented with non-essential amino acids, glutamine and 5% heat-inactivated fetal bovine serum [19]. This formulation is called E-5 medium.

#### Genomic DNA preparation

Cells grown as suspended vesicles for 4 to 5 days in twenty 60 mm plates were collected by centrifugation, and the cell pellet was washed twice with phosphate-buffered saline (PBS; [20]). The cell pellet was resuspended in 20 ml lysis buffer (10 mM Tris-HCl, pH 7.5, 10 mM EDTA, 200 μg/ml proteinase K), and SDS was added to a final concentration of 0.5%. The lysate was incubated at 37°C overnight. NaCl was added to a final concentration of 0.4 M, and the DNA was extracted once with 20 ml phenol, twice with an equal volume of phenol:chloroform (1:1), and twice with an equal volume of chloroform. Two volumes of ethanol were added, and DNA was spooled onto a clean glass rod. The DNA was dried, and dissolved in 10 ml of TE (10 mM Tris-HCl, pH 8.0, containing 1 mM EDTA) at 37°C. RNase A was added to a final concentration of 200 μg/ml and incubated at 37°C for 4 hours. DNA was phenol extracted, ethanol precipitated and dissolved in TE as described above.

#### DNA amplification by PCR

Genomic DNA (0.4 mg) was digested with *Hin*dIII (Promega) at 37°C overnight. Enzyme was removed by phenol:choloroform extraction, and the DNA was recovered by precipitation with ethanol and dissolved in TE. Digested DNA (100 ng) was used as template for the PCR reaction, which contained 1X PCR buffer, 1.5 mM MgCl_2_, 0.2 mM of each of the four dNTPs, 0.4 μM of primer F1 (5'CCG AAG AAG CCG AAG AAG CCC) and R1 (5'TGC TTT CGG CTT CTT GGC AGC) and 2.5 units of Taq DNA polymerase (Promega, Madison, WI). PCR was performed with an initial denaturation at 94°C for 2 minutes. The next 35 cycles included 94°C denaturation for 45 sec, 55°C annealing for 1 minute, and 72°C extension for 1 minute. The reaction was terminated by a final elongation cycle at 72°C for 2 minutes. The PCR product was recovered from a 0.9% agarose gel, purified using Ultra-Clean 15 (MO Bio Laboratories Inc., Solana Beach, CA) and cloned into PGEM T-Easy vector (Promega). The 3'-end of the gene was obtained in a similar manner, using primers R2 (Fig. 2) and F1.

#### Amplifying the 5'-end of the cDNA

Total RNA was recovered from ASE-IV cells by guanidine isothiocyanate extraction and cesium chloride centrifugation as described by Davis et al. [21]. The final RNA pellet was dissolved in DEPC-treated water and stored at -70°C. RNA (1 μg) was used with the GeneRacer kit (Invitrogen) to obtain the 5' end of the mRNA, using primer R1 as the reverse primer.

#### Programs and accession numbers

The analysis in Fig. 4A was produced using the Genetics Computer Group (GCG; Madison, WI) program "gap". The tree in Fig. 4B and the alignment in Fig. 5 were produced by an alignment of amino acid residues using default parameters of Clustal X (version 1.83) [22]. The tree was created in PAUP* [23], with the *C. elegans *H1 protein designated as an outgroup. The *An. stephensi *histone H1 sequence has GenBank accession # AY672907.

## Authors' contributions

YZ did the experimental work, AMF helped with experimental design and manuscript preparation. Both authors read and approved the final manuscript.
